# Editorial for the Special Issue “New Research in Childhood Nutrition” of the Journal *Children*

**DOI:** 10.3390/children11030375

**Published:** 2024-03-21

**Authors:** Jose Joaquín Muros

**Affiliations:** Department of Didactics of Corporal Expression, University of Granada, 18071 Granada, Spain; jjmuros@ugr.es

Nutrition is much more than food. Consumption of a balanced and nutritional diet may have positive effects on physical, behavioural and psychosocial factors. Childhood is a key stage for the acquisition of behaviours that are likely to persist into adulthood. Healthy eating habits have been demonstrated to have a positive influence on preventing chronic diseases and improving mental health in children and adolescents. This Special Issue provides new and novel approaches to identify gaps in nutritional knowledge related with children’s health.

Dietary guidelines refer to food choices and dietary patterns that meet requirements for essential nutrients and protect against chronic diseases. Five papers cover subjects related to this topic. Chen et al. (Contribution 1) validate previous structural models comprising factors that are related with dietary behaviour change and, subsequently, construct models to predict knowledge, attitudes and behaviour in school footballers in China. McEachern et al. (Contribution 2) investigate factors associated with fruit and vegetable consumption in elementary school children in Ontario, Canada. Camaño-Navarrete et al. (Contribution 3) determine the association of selective attention and concentration with physical fitness, lifestyle parameters and anthropometric measures among Chilean schoolchildren. Pérez-Marmol et al. (Contribution 4) analyse the relationships between physical self-concept, physical activity engagement and Mediterranean diet adherence in a sample of secondary school students in Granada. Melguizo-Ibáñez et al. (Contribution 5) describe the degree of family functionality, emotional intelligence, Mediterranean diet adherence, and extra-curricular physical activity engagement in Granada, Spain.

Adequate nutrition and food security is essential for ensuring growth and optimal development, whilst, at the same time, preventing chronic disease. Two papers are presented in relation to this topic. Dubelt-Moroz et al. (Contribution 6) investigate food security, dietary intake and eating behaviour in a convenience sample of young people participating in the Maple Leaf Sports Entertainment LaunchPad program in downtown Toronto, Ontario. Garro-Mellado et al. (Contribution 7) provide information on sugars in the formulation of cereals marketed towards infants.

A transition has taken place from more traditional diets to diets that are high in sugar and salt. Although there are multiple causes of this pattern change, advertising can be considered as one of the main triggers. This change is related with greater obesity and chronic disease. In relation to this topic, Montaña-Blasco (Contribution 8) analyse breakfast product advertisements as a function of their target audience, use of celebrities or popular figures/characters to encourage purchase and product sugar content. Campos et al. (Contribution 9) analyse food advertising aimed towards children and disseminated through Spanish television in 2013 and 2018 on children’s and general channels, focusing on the effect of laws and statutory requirements over time.

Obesity is considered to be an issue inherent to high-income countries. However, overweight and obesity are now on the rise in low- and middle-income countries, particularly in urban settings. Obesity is a major risk factor for non-communicable diseases. Two papers cover topics related to this subject. Monyeki et al. (Contribution 10) determine the association between body weight and body composition derived from deuterium oxide (D2O) dilution in 6-to-8-year-old South African children. Naqiah Zulkifli et al. (Contribution 11) determine the association between atypical mealtime behaviours and associated risk factors, whilst also exploring the prevalence of overweight and obesity in Malaysian children with autism spectrum disorder in community settings.

Two papers from this Special Issue explore selected issues related with infant feeding practices. Ruangkit et al. (Contribution 12) evaluate the association of infant feeding practices in the first 6 months of life with iron status and hematologic parameters. Modjadji et al. (Contribution 13) determine the association of maternal tobacco and alcohol use with malnutrition indicators among infants in Gauteng, South Africa.

Two papers from this Special Issue explore selected issues related with lifestyle and COVID. Children all over the world were affected by confinement measures. During the pandemic period, it was difficult to avoid engaging in sedentary habits, whilst, at the same time, the consumption of calorie-dense foods increased due to anxiety, stress and boredom. Busto-Arriagada et al. (Contribution 14) evaluate compliance with lifestyle recommendations proposed at both national and international levels in children aged 0 to 23 months during confinement resulting from the COVID-19 pandemic in Chile. Villodres et al. (Contribution 15) study the influence of lockdown due to COVID-19 on physical activity engagement and Mediterranean diet adherence and, subsequently, the impact of this on self-esteem in pre-adolescent students in Spain.

Three more papers are presented that consider various subjects, such as bone metabolism, in which Klentrou et al. (Contribution 16) examine the effects of Greek yogurt consumption on bone biomarkers during 5 days of intense soccer training. Next, Song et al. (Contribution 17) investigate the association of dyslipidemia with vitamin D and physical activity in Korean children and adolescents. Finally, Bennet et al. (Contribution 18) present a review of school-based culinary interventions, in which they describe the characteristics of school-based culinary interventions ran during school hours and determine the contribution of these for child health outcomes.

I hope that this Special Issue will serve as a repository of knowledge for paediatricians and nutritionists seeking to conduct further research in the field. It also aims to help professionals working with children and adolescents explore the way in which different nutritional strategies may impact upon physical and mental health during these stages of childhood.

